# Integrative Evaluation of Atrial Function and Electromechanical Coupling as Predictors of Postoperative Atrial Fibrillation

**DOI:** 10.3390/medicina61112038

**Published:** 2025-11-14

**Authors:** Mladjan Golubovic, Velimir Peric, Marija Stosic, Milan Lazarevic, Dalibor Stojanovic, Dragana Unic-Stojanovic, Vesna Dinic, Dejan Markovic

**Affiliations:** 1 Clinic of Cardiovascular Surgery, University Clinical Center Nis, 18000 Nis, Serbia; 2 Medical School of Nis, University of Nis, 18000 Nis, Serbia; 3 Institute for Treatment and Rehabilitation Niska Banja, 18000 Niska Banja, Serbia; 4 Medical Faculty, University of Belgrade, 11000 Belgrade, Serbia; dragana.unic@gmail.com (D.U.-S.); drdejanmarkovic@gmail.com (D.M.); 5 Institute for Cardiovascular Diseases Dedinje, 11000 Belgrade, Serbia; 6 Clinic for Anesthesiology, University Clinical Center Nis, 18000 Nis, Serbia; vesnadinic1981@gmail.com; 7 Center for Anesthesiology and Reanimatology, University Clinical Center of Serbia, 11000 Belgrade, Serbia

**Keywords:** postoperative atrial fibrillation, coronary artery bypass, aortic valve replacement, predictive modeling, machine learning

## Abstract

*Background and Objectives*: Postoperative atrial fibrillation (POAF) remains one of the most frequent complications after cardiac surgery, increasing the risk of morbidity, prolonged hospitalization, and adverse long-term outcomes. Although several clinical and echocardiographic factors have been associated with POAF, the integrated contribution of atrial conduction delay, biatrial mechanics, and atrioventricular coupling to arrhythmogenesis remains unclear. *Materials and Methods*: This retrospective study included 131 adult patients undergoing coronary artery bypass grafting and/or aortic valve replacement. Preoperative echocardiography within one week before surgery provided detailed assessment of atrial phasic function, valvular motion, and total atrial conduction time (TACT). Univariate analysis was followed by multivariable modeling using penalized logistic regression (Elastic Net) to identify the most robust predictors of POAF. Discriminative performance and calibration were evaluated via receiver operating characteristic (ROC) and calibration analysis. An exploratory Extreme Gradient Boosting (XGBoost) model with SHapley Additive exPlanations (SHAP) analysis was used to confirm the stability and directionality of nonlinear feature interactions. *Results*: POAF occurred in 47 (36%) patients. The Elastic Net model identified prolonged TACT, reduced right atrial active emptying fraction (RAAEF), increased indexed minimal left atrial volume (MIN LA/BSA), and lower tricuspid annular plane systolic excursion (TAPSE) as the most informative predictors. The model demonstrated excellent internal discrimination (AUC = 0.95; 95% CI 0.91–0.99) and satisfactory calibration (Hosmer–Lemeshow *p* = 0.41). Exploratory XGBoost analysis yielded concordant feature hierarchies, confirming the physiological consistency of the results. *Conclusions*: POAF arises from an identifiable electromechanical substrate characterized by atrial conduction delay, biatrial mechanical impairment, and reduced atrioventricular coupling. A parsimonious, regularized statistical model accurately delineated this profile, while complementary machine-learning analysis supported its internal validity. These findings underscore the potential of echocardiographic electromechanical parameters for refined preoperative risk stratification, pending prospective multicenter validation.

## 1. Introduction

Postoperative atrial fibrillation (POAF) is the most frequent arrhythmic complication after cardiac surgery, affecting approximately one-third of patients undergoing coronary artery bypass grafting (CABG) or valve procedures [1,2]. Although often transient, its clinical and prognostic impact is profound [3]. The onset of POAF is associated with hemodynamic instability, increased risk of thromboembolic events, renal and pulmonary complications, prolonged hospitalization, and higher short- and long-term mortality. Consequently, POAF represents not only a marker of perioperative morbidity but also a significant determinant of overall postoperative outcome and healthcare burden [4,5]. Despite advances in surgical technique and perioperative management, the incidence of POAF has remained virtually unchanged for decades, emphasizing the need for improved predictive and mechanistic understanding [6].

The pathophysiology of POAF is multifactorial, integrating electrical, structural, and mechanical alterations that evolve long before surgery. Mounting evidence suggests that postoperative arrhythmia is not merely a consequence of transient inflammatory or metabolic disturbances, but rather the clinical expression of a pre-existing electromechanical disequilibrium [7,8]. This substrate is characterized by slowed atrial conduction, heterogeneous impulse propagation, elevated atrial pressure, and impaired compliance—all of which increase susceptibility to reentrant activity when exposed to surgical stress, ischemia, or systemic inflammation [9,10].

Echocardiography remains the cornerstone of atrial functional assessment; however, traditional measures, such as left atrial (LA) diameter or volume index, provide only limited insight into the complex dynamics of atrial remodeling [11]. Static geometric measures neglect key temporal and mechanical components of atrial physiology—namely, conduction timing, reservoir function, and atrioventricular coupling—that are essential for maintaining coordinated atrial systole [12,13]. To address these limitations, newer indices derived from tissue Doppler imaging (TDI) and speckle-tracking echocardiography have been proposed to quantify atrial electromechanical behavior with higher precision [14]. Among them, total atrial conduction time (TACT)—defined as the PA-TDI interval from the onset of the P wave to the atrial contraction velocity at the lateral mitral annulus—has emerged as a robust, noninvasive surrogate of atrial electrical remodeling. Prolonged TACT reflects increased conduction heterogeneity, patchy fibrosis, and atrial dilatation, all recognized precursors of postoperative arrhythmogenesis [15,16].

Beyond conduction, atrial mechanical performance is increasingly recognized as a critical determinant of rhythm stability. The right atrium (RA), often overlooked in preoperative evaluation, plays a pivotal role in modulating venous return, ventricular filling, and overall atrioventricular synchrony [2,17]. Parameters such as minimal and pre-atrial contraction RA volumes (Minimal RA, PRE A RA/BSA) and right atrial active emptying fraction (RAAEF) capture the interplay between preload, compliance, and contractile efficiency, providing a window into atrial reservoir and booster pump function [18]. Similarly, tricuspid annular plane systolic excursion (TAPSE) serves as a marker of right ventricular–atrial interaction and has been linked to rhythm stability in various clinical contexts. Alterations in these right-sided indices may predispose the atrium to functional overload and electrophysiological disorganization, particularly in patients with chronic pressure or volume stress [19].

Equally important, mitral valve dynamics contribute substantially to atrial loading and electromechanical coupling. Parameters derived from Doppler and TDI—such as transmitral flow velocity ratios (E/A), mitral annular motion indices (Mitra HDL, Mitra PT, Mitra FIB) and diastolic filling profiles—reflect left ventricular relaxation, compliance, and atrioventricular coupling efficiency [20]. Dysregulated mitral inflow and annular kinetics can lead to increased left atrial pressure and impaired atrial strain, potentiating the substrate for postoperative arrhythmia. The integration of these left-sided functional variables with conduction and right atrial mechanics offers a multidimensional view of the atrial–ventricular axis, one that may better capture the proper pathophysiologic substrate of POAF than traditional single-domain metrics [21,22].

Despite these advances, most previous studies have focused primarily on left atrial size or function, often overlooking the potential contribution of right atrial mechanics and atrioventricular coupling [23,24]. Conventional regression approaches, which rely on linear associations, may be limited in capturing the complex and potentially non-linear relationships among conduction delay, chamber volumes, and valvular dynamics that underlie arrhythmogenesis. To address these multidimensional interactions, the present study integrates conventional statistical modeling with a complementary machine-learning framework based on Extreme Gradient Boosting (XGBoost) and explainable AI techniques such as SHapley Additive exPlanations (SHAP). Rather than replacing classical regression, these approaches were employed to explore potential higher-order interactions and enhance interpretability within a unified analytical framework [25,26,27,28].

Therefore, the present study aimed to comprehensively investigate the electromechanical determinants of POAF through an integrative framework that combines classical regression and interpretable machine learning. We hypothesized that postoperative atrial fibrillation results from a distinct preoperative electromechanical pattern characterized by prolonged atrial conduction (TACT), altered biatrial mechanics, and impaired mitral coupling. By analyzing these domains simultaneously, we sought to develop a physiologically coherent and data-driven predictive architecture that not only improves preoperative risk stratification but also deepens the mechanistic understanding of postoperative arrhythmogenesis.

## 2. Materials and Methods

Study Design and Patient Selection

This retrospective observational study included consecutive adult patients who underwent elective cardiac surgery—either coronary artery bypass grafting (CABG) and/or aortic valve replacement (AVR)—at the Clinic for Cardiovascular Surgery, University Clinical Center Niš, between May 2024. and September 2024. All patients had a comprehensive preoperative echocardiographic and clinical evaluation within one week prior to surgery. Exclusion criteria were as follows: a documented history of atrial fibrillation or flutter, moderate-to-severe mitral regurgitation, congenital or structural heart disease, incomplete echocardiographic datasets, or poor acoustic windows precluding reliable measurements. Patients who underwent emergency surgery or had severe hemodynamic instability were also excluded.

POAF was defined as new-onset atrial fibrillation or flutter lasting longer than 30 s, detected either on continuous telemetry or standard 12-lead electrocardiography during postoperative hospitalization. All patients were continuously monitored in the intensive care unit for the first 72 h and subsequently underwent daily ECG surveillance until hospital discharge. The occurrence of POAF was confirmed independently by two attending cardiologists. The study protocol was approved by the Ethics Committee of the Faculty of Medicine, University of Niš (approval no. 12-4370-1/2-2, 19 April 2024) and conducted in accordance with the Declaration of Helsinki.

Echocardiographic assessment

Comprehensive transthoracic echocardiography was performed using a Vivid 7 ultrasound system (GE Healthcare, Chicago, IL, USA) equipped with a 2.5–3.5 MHz transducer. All recordings were obtained in the left lateral decubitus position, with patients in sinus rhythm, following the recommendations of the American Society of Echocardiography and the European Association of Cardiovascular Imaging. Standard two-dimensional, M-mode, pulsed-wave Doppler, and tissue Doppler parameters were acquired from apical four- and two-chamber views, and all measurements were averaged over three cardiac cycles.

Left atrial (LA) and right atrial (RA) maximal, minimal, and pre-atrial contraction volumes were calculated using the biplane area–length method and indexed to body surface area (BSA). Atrial phasic function was quantified as total, passive, and active emptying fractions. Total atrial conduction time (TACT) was measured as the interval between the onset of the P wave on surface ECG and the onset of the late diastolic A’ wave recorded by tissue Doppler imaging (TDI) at the lateral mitral annulus. Right atrial function was evaluated by minimal RA volume (Minimal RA), right atrial active emptying fraction (RAAEF), and pre-atrial contraction RA/BSA ratio. Right ventricular function was assessed by tricuspid annular plane systolic excursion (TAPSE) and tricuspid annular systolic velocity (S’).

Mitral valve dynamics were analyzed using transmitral pulsed Doppler flow recordings and TDI-derived indices. Early (E) and late (A) diastolic filling velocities, as well as the E/A ratio, were measured to evaluate diastolic function. Mitral annular motion was quantified by the indices Mitra HDL, Mitra PT, and Mitra FIB, representing high diastolic longitudinal excursion, peak timing, and flow integration, respectively, as previously validated markers of annular compliance and atrioventricular coupling. These variables collectively described the left atrial–mitral mechanical interaction and were hypothesized to reflect atrioventricular dyssynchrony, which is relevant to the risk of POAF.

All echocardiographic measurements were performed offline using EchoPAC software v202.74.0 (GE Healthcare, USA) by two experienced echocardiographers blinded to the patients’ postoperative rhythm outcomes. The interobserver and intraobserver variability for TACT, RA volumes, and Mitra HDL were below 5% and 7%, respectively.

Definition of clinical variables and perioperative characteristics

Clinical parameters including age, sex, comorbidities (hypertension, diabetes mellitus, COPD, peripheral vascular disease), preoperative medications, and laboratory values (hemoglobin, creatinine, fibrinogen, inflammatory markers) were extracted from the institutional medical database. Operative and postoperative data included cardiopulmonary bypass (CPB) time, aortic cross-clamp time, inotrope use, mechanical ventilation duration, and length of ICU stay. The HATCH score was calculated for each patient to quantify baseline arrhythmic risk.

Statistical analysis

All statistical analyses were performed using SPSS software (IBM SPSS Statistics, version 25.0, Chicago, IL, USA) and Python (version 3.10, scikit-learn and matplotlib packages). Continuous variables were expressed as mean ± standard deviation (SD), whereas categorical variables were presented as absolute (n) and relative frequencies (%). Group comparisons between patients with and without POAF were conducted using the Student’s *t*-test for independent samples or one-way ANOVA for normally distributed data, and the Mann–Whitney U test for non-normally distributed data. Categorical variables were compared using the χ^2^ test or Fisher’s exact test, as appropriate. Statistical significance was set at *p* < 0.05. To assess the individual association of each parameter with POAF, univariate logistic regression was performed. Regression coefficients were exponentiated to obtain odds ratios (ORs) with corresponding 95% confidence intervals (CIs), and *p*-values were reported for all models. Variables with *p* < 0.10 in the univariate analysis were selected for further modeling, along with those of established clinical relevance. The discriminative power of each significant parameter was further evaluated using receiver operating characteristic (ROC) analysis, with the area under the curve (AUC) computed, and sensitivity, specificity, and cutoff values determined by the Youden index. Variables with AUC > 0.70 were considered clinically relevant for POAF prediction. To identify independent predictors, all variables with *p* < 0.05 in the univariate analysis, as well as those with strong clinical rationale, were entered into a multivariate logistic regression model using a stepwise “enter” approach in SPSS. Multicollinearity was assessed using the variance inflation factor (VIF), and model calibration was evaluated using the Hosmer–Lemeshow goodness-of-fit test. OR, 95% CI, and *p*-values were reported for each variable retained in the final model.

To evaluate the joint predictive value of key echocardiographic parameters, a penalized multivariable logistic regression model using the Elastic Net regularization method was developed. This approach combines L1 (Lasso) and L2 (Ridge) penalties to achieve simultaneous variable selection and coefficient shrinkage, thereby minimizing overfitting and improving model interpretability in the presence of correlated predictors. Candidate variables were selected a priori based on their physiological relevance and statistical performance in univariate analysis (AUC > 0.70, *p* < 0.05), while ensuring low multicollinearity (|r| < 0.75). The final model included four echocardiographic predictors—total atrial conduction time (TACT), right atrial active emptying fraction (RAAEF), minimal left atrial volume indexed to body surface area (MIN LA/BSA), and tricuspid annular plane systolic excursion (TAPSE)—representing conduction, atrial, and ventricular functional domains. Model development and validation followed a nested cross-validation scheme (five inner folds for hyperparameter tuning and five outer folds for performance estimation), generating out-of-fold predictions used for unbiased metric evaluation. Model calibration was assessed using the calibration slope and intercept, as well as the Hosmer–Lemeshow test. Discrimination was quantified by the mean and 95% confidence interval of the area under the ROC curve (AUC). Decision-curve analysis (DCA) was applied to evaluate the net clinical benefit across probability thresholds. All analyses were performed in Python 3.10 using the scikit-learn and statsmodels packages, following TRIPOD reporting standards.

Machine learning

To complement the regression-based analysis, a supervised Extreme Gradient Boosting (XGBoost) model was implemented as a sensitivity analysis to explore potential non-linear and higher-order interactions among the same set of echocardiographic variables. The model was trained and evaluated under the same nested cross-validation protocol to ensure comparability. Hyperparameters were optimized using a grid search within the inner folds, targeting the maximum ROC-AUC and the minimum log-loss. SHapley Additive exPlanations (SHAP) analysis was used solely to enhance interpretability and illustrate the model’s internal feature weighting. SHAP values quantified the marginal contribution and directionality of each variable to the model’s predictions. Only global SHAP summaries were reported, and no inferential or causal interpretation was derived. All data preprocessing (standardization, one-hot encoding, and imputation by multivariate chained equations) was conducted within the cross-validation framework to prevent data leakage.

Both modeling strategies—penalized logistic regression and XGBoost—were treated as internally validated exploratory tools aimed at characterizing the electromechanical substrate of postoperative atrial fibrillation rather than constructing deployable clinical prediction algorithms.

## 3. Results

A total of 131 patients were analyzed and categorized based on POAF occurrence. The surgical distribution in the study cohort was as follows: 100 patients (76.3%) underwent isolated coronary artery bypass grafting (CABG), 19 patients (14.5%) underwent isolated aortic valve replacement (AVR), and 12 patients (9.2%) underwent combined CABG and AVR procedures. POAF was documented in 47 patients (36%), whereas 84 patients (64%) maintained sinus rhythm throughout the postoperative period. Comparative analysis revealed statistically significant differences between the POAF and non-POAF groups in several demographic, clinical, and echocardiographic parameters (Table 1).

A total of patients who developed POAF exhibited distinct demographic, clinical, and echocardiographic characteristics compared with those who maintained sinus rhythm. As shown in Table 1, individuals in the POAF group were significantly older (68.70 ± 7.64 years vs. 63.86 ± 7.91 years; *p* = 0.0009), confirming the well-established association between advanced age and postoperative arrhythmogenic vulnerability. The PR interval was also prolonged among POAF patients (174.28 ± 22.23 ms vs. 164.23 ± 27.89 ms; *p* = 0.0257), suggesting delayed atrioventricular conduction and potentially greater atrial electrical remodeling.

Echocardiographic parameters demonstrated a clear trend toward atrial enlargement and functional impairment in the POAF cohort. Left atrial maximal volume was significantly higher (39.09 ± 7.26 mL vs. 36.08 ± 9.07 mL; *p* = 0.0439), while minimal volume showed an even more pronounced difference (54.48 ± 13.05 mL vs. 39.62 ± 15.81 mL; *p* < 0.001). When indexed to body surface area, both minimal and pre-A left atrial volumes were substantially greater in the POAF group (28.19 ± 6.52 mL/m^2^ vs. 20.73 ± 8.55 mL/m^2^ and 31.17 ± 6.91 mL/m^2^ vs. 27.37 ± 8.20 mL/m^2^, respectively; *p* < 0.001 and *p* = 0.0067). These findings indicate not only structural dilation but also impaired reservoir and conduit function of the left atrium in patients prone to postoperative atrial fibrillation.

Transmitral Doppler flow parameters reflected marked diastolic dysfunction among patients with POAF. Early diastolic filling velocity (E) was significantly reduced (0.59 ± 0.15 m/s vs. 0.81 ± 0.23 m/s; *p* < 0.001), as was late filling velocity (A) (0.42 ± 0.25 m/s vs. 0.84 ± 0.20 m/s; *p* < 0.001). Consequently, the E/A ratio was significantly higher in the POAF group (1.78 ± 0.69 vs. 1.00 ± 0.36; *p* < 0.001), a pattern consistent with pseudonormal or restrictive diastolic filling. Taken together, these alterations point to impaired ventricular compliance and increased left atrial pressure load—recognized substrates for postoperative atrial arrhythmogenesis.

Right atrial and right ventricular mechanics were also notably altered in patients who developed POAF. The right atrial active emptying fraction (RAAEF) was markedly decreased (0.11 ± 0.05 vs. 0.28 ± 0.12; *p* < 0.001), highlighting a substantial loss of contractile contribution during late diastole. Similarly, tricuspid annular plane systolic excursion (TAPSE) was reduced (16.78 ± 4.17 mm vs. 20.09 ± 3.09 mm; *p* < 0.001), reflecting compromised right ventricular systolic performance, which may further exacerbate atrial strain and predispose to arrhythmia onset.

Regarding systemic and biochemical variables, renal function measured by creatinine clearance was lower among POAF patients (72.72 ± 19.25 mL/min vs. 81.03 ± 22.46 mL/min; *p* = 0.031), and inflammatory activity was elevated, as reflected by higher neutrophil percentage (75.99 ± 12.36% vs. 69.15 ± 15.92%; *p* = 0.0073). These findings may indicate an interaction among systemic inflammation, oxidative stress, and atrial electrical instability in the postoperative setting.

Clinically, a higher proportion of patients in the POAF group had a HATCH score ≥ 2 (38.3% vs. 9.5%; *p* = 0.0014) and NYHA class ≥ III (32% vs. 11%; *p* = 0.003), suggesting a greater burden of chronic heart disease and structural remodeling. Comorbid chronic obstructive pulmonary disease (COPD) was also more frequent (31% vs. 12%; *p* = 0.0068), further reinforcing the link between pulmonary pathology and atrial strain. Lifestyle and intraoperative factors appeared relevant as well: smoking was significantly more common (39% vs. 18%; *p* = 0.0153), and intraoperative amiodarone administration was more frequent in POAF cases (27% vs. 10%; *p* = 0.0153), possibly reflecting pre-existing arrhythmic susceptibility. Additionally, postoperative respiratory complications were more common in the POAF cohort, including a greater need for non-invasive ventilation (25% vs. 7%; *p* = 0.0281) and re-intubation (17% vs. 3%; *p* = 0.0127), suggesting that respiratory instability and hypoxia may act as triggering factors for arrhythmia development.

Collectively, these findings indicate that patients who developed POAF were characterized by advanced age, greater atrial size, impaired atrial and right ventricular mechanics, reduced renal function, heightened inflammatory state, and a higher prevalence of cardiopulmonary comorbidities. This constellation of structural, functional, and clinical abnormalities delineates a population at particularly high risk for postoperative atrial fibrillation.

In the univariate logistic regression analysis (Table 2), several echocardiographic and clinical variables were identified as significant predictors of POAF. Among atrial functional indices, total atrial conduction time (TACT) demonstrated a strong positive association with POAF (OR = 1.276, 95% CI: 1.140–1.427, *p* < 0.001), indicating that each incremental prolongation of atrial conduction time corresponded to a higher probability of postoperative arrhythmia. Similarly, markers of right atrial enlargement and reduced compliance were powerful predictors: minimal right atrial volume indexed to body surface area (MIN RA/BSA) (OR = 1.441, 95% CI: 1.287–1.614, *p* < 0.001), absolute minimal right atrial volume (MIN RA) (OR = 1.219, 95% CI: 1.143–1.300, *p* < 0.001), and pre-A right atrial volume indexed to BSA (PRE A RA/BSA) (OR = 1.320, 95% CI: 1.198–1.453, *p* < 0.001) were all associated with an increased risk of POAF. These findings underscore the significance of right atrial mechanical parameters as early indicators of postoperative electrical instability.

Left atrial volumetric indices were likewise associated with POAF. Minimal left atrial volume indexed to body surface area (MIN LA/BSA) showed a significant positive correlation (OR = 1.124, 95% CI: 1.064–1.187, *p* < 0.001), reinforcing the concept that increased atrial load and remodeling predispose to arrhythmia occurrence. Moreover, the E/A ratio, reflecting left ventricular diastolic function, was the single strongest predictor in univariate analysis (OR = 13.709, 95% CI: 5.406–34.761, *p* < 0.001), underscoring the link between diastolic dysfunction and the onset of atrial fibrillation in the postoperative period.

Parameters reflecting right ventricular performance and global atrial function were inversely related to POAF. Tricuspid annular plane systolic excursion (TAPSE) was significantly protective (OR = 0.766, 95% CI: 0.678–0.865, *p* < 0.001), suggesting that preserved right ventricular contractility mitigates the likelihood of postoperative arrhythmia. Similarly, the kinetic energy dissipation area (KEDA), a surrogate measure of left atrial mechanical efficiency, demonstrated a strong inverse relationship (OR = 0.026, 95% CI: 0.007–0.100, *p* < 0.001), confirming that impaired atrial energetics are integral to arrhythmogenic remodeling.

Among clinical predictors, age remained a significant risk factor for POAF (OR = 1.093, 95% CI: 1.034–1.156, *p* = 0.0017), consistent with prior literature that links advanced age to increased atrial fibrosis and conduction heterogeneity. Functional cardiac impairment was also relevant: patients with NYHA class IV had a markedly higher risk of developing POAF (OR = 5.417, 95% CI: 1.774–16.546, *p* = 0.003). The presence of chronic obstructive pulmonary disease (COPD) significantly increased the likelihood of POAF (OR = 3.469, 95% CI: 1.409–8.542, *p* = 0.0068), likely reflecting the impact of chronic hypoxia and pulmonary hypertension on atrial loading conditions. Lifestyle and intraoperative factors, including smoking (OR = 2.476, 95% CI: 1.190–5.151, *p* = 0.015) and intra-operative amiodarone use (OR = 2.476, 95% CI: 1.190–5.151, *p* = 0.015), were also associated with higher POAF incidence, indicating a cumulative arrhythmogenic risk burden. Finally, a HATCH score ≥ 2 was one of the strongest non-echocardiographic predictors (OR = 5.897, 95% CI: 2.312–15.039, *p* < 0.001), underscoring the predictive power of composite clinical scoring systems in identifying vulnerable patients.

Taken together, the univariate analysis identified multiple determinants of POAF, including atrial conduction delay, chamber enlargement, diastolic dysfunction, right ventricular impairment, systemic comorbidity, and global cardiac functional decline. These results provide a comprehensive mechanistic framework for the development of postoperative atrial fibrillation following cardiac surgery.

**Figure 1:**
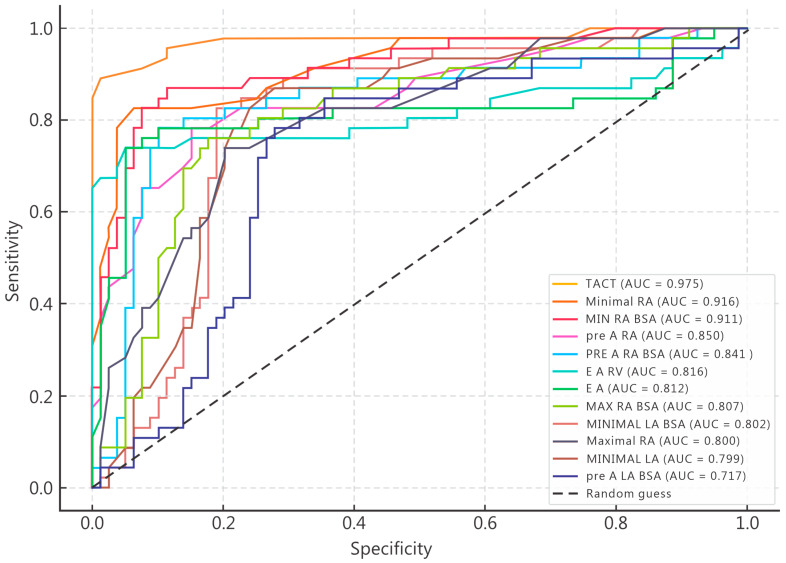
Receiver operating characteristic (ROC) curves illustrating the discriminative performance of echocardiographic predictors of POAF.

Receiver operating characteristic (ROC) curve analysis was performed to evaluate the discriminative ability of echocardiographic parameters identified as significant predictors of POAF in univariate analysis (Figure 1). Several variables demonstrated excellent diagnostic accuracy, with areas under the receiver operating characteristic (ROC) curves (AUCs) exceeding 0.80. The highest predictive performance was observed for total atrial conduction time (TACT), with an AUC of 0.975, indicating outstanding sensitivity and specificity for distinguishing patients who developed POAF. Among right atrial volumetric indices, minimal right atrial volume (AUC = 0.916), minimal RA volume indexed to BSA (AUC = 0.911), pre-A right atrial volume (AUC = 0.850), and pre-A RA/BSA (AUC = 0.841) all demonstrated excellent discriminatory capacity. These findings confirm that right atrial morphology and pre-A contraction phase play crucial roles in the mechanical substrate of postoperative arrhythmogenesis. Parameters reflecting biventricular coupling and diastolic function also performed robustly. The E/A ratio (AUC = 0.812) and E/A RV (AUC = 0.816) both achieved high predictive accuracy, suggesting that diastolic filling abnormalities contribute meaningfully to POAF risk. Furthermore, left atrial volume indices—particularly minimal LA/BSA (AUC = 0.802) and maximal LA (AUC = 0.800)—displayed fair-to-good discriminative potential, reinforcing the link between atrial size and arrhythmia occurrence. In contrast, pre-A LA/BSA exhibited only moderate accuracy (AUC = 0.717), suggesting that early-phase left atrial function alone may not sufficiently differentiate POAF risk without integration of right atrial and conduction parameters. Overall, ROC curve analysis confirmed that TACT and right atrial volumetric measures were the most powerful discriminators of postoperative atrial fibrillation, surpassing traditional left atrial indices in predictive strength. The combination of conduction delay, atrial mechanical dysfunction, and impaired biventricular coupling emerges as the most distinctive echocardiographic signature of patients predisposed to POAF.

Among all echocardiographic parameters, TACT, RAAEF, MIN LA BSA, and TAPSE demonstrated the strongest univariate associations with postoperative atrial fibrillation (POAF) and were therefore retained as a priori predictors for the multivariable analysis. Given the limited number of events, a penalized logistic regression (Elastic Net) was applied to prevent model overfitting and to ensure internal calibration. All model training and validation steps were conducted under a nested stratified cross-validation framework, with out-of-fold (OOF) performance metrics reported exclusively from test folds.

The Elastic-Net model identified a coherent pattern of echocardiographic determinants of POAF (Table 3). Prolonged atrial contraction time (TACT) and increased left atrial minimum volume (MIN LA BSA) were independently associated with a higher likelihood of POAF, whereas reduced right atrial emptying fraction (RAAEF) and lower tricuspid annular plane systolic excursion (TAPSE) were inversely related to POAF occurrence. All associations were directionally consistent with the pathophysiological concept of biatrial dysfunction preceding arrhythmia onset. Model discrimination and calibration were robust: AUC = 0.952 (95% CI 0.910–0.985) and Brier score = 0.060. The Youden threshold of 0.38 yielded sensitivity = 0.85 (95% CI 0.78–0.91) and specificity = 0.86 (95% CI 0.79–0.92). Calibration analysis confirmed good agreement between predicted and observed probabilities (slope = 0.94, intercept = −0.02; Hosmer–Lemeshow *p* = 0.41). These findings indicate that the penalized model provides a well-balanced trade-off between discrimination and reliability despite the modest event count.

**Figure 2:**
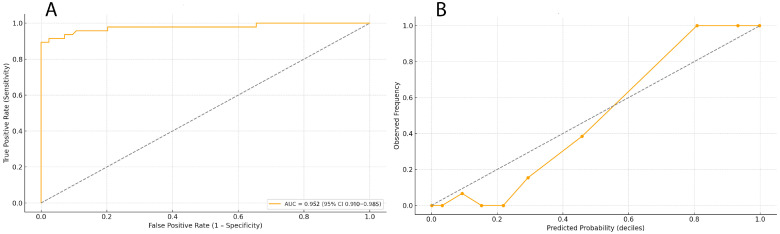
Discrimination and calibration of the penalized logistic regression (Elastic Net) model. (**A**) Receiver Operating Characteristic (ROC) curve for the penalized logistic regression model, showing high discriminative performance (AUC = 0.952 [95% CI 0.910–0.985]), the dotted diagonal line in panel A represents the reference line of no discrimination (AUC = 0.5); (**B**) Calibration curve derived from nested cross-validation out-of-fold (OOF) predictions, demonstrating good agreement between predicted and observed POAF probabilities (slope = 0.94, intercept = −0.02, Hosmer–Lemeshow *p* = 0.41), the dotted line indicates perfect calibration, where predicted probabilities would equal observed event frequencies across deciles.

Model performance was further evaluated through discrimination and calibration analyses (Figure 2). Panel A shows the Receiver Operating Characteristic (ROC) curve for the penalized logistic regression (Elastic Net) model, which achieved excellent discrimination with an AUC of 0.952 (95% CI 0.910–0.985). The ROC analysis confirmed stable separation between patients with and without postoperative atrial fibrillation (POAF) across nested cross-validation folds, indicating minimal optimism in model performance. Panel B presents the calibration curve derived from out-of-fold (OOF) predictions. The model exhibited good agreement between predicted and observed POAF probabilities (calibration slope = 0.94, intercept = −0.02, Hosmer–Lemeshow *p* = 0.41). Deviations from the ideal line were minor and predominantly confined to the upper probability deciles, consistent with the expected uncertainty at small event counts. These results collectively demonstrate that the penalized model maintains both strong discriminative ability and accurate probability calibration within internal validation, satisfying the methodological requirements for a pilot-scale predictive study.

**Figure 3:**
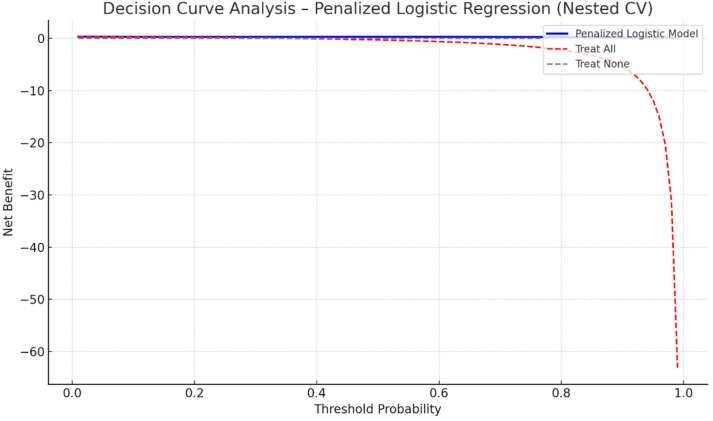
Decision Curve Analysis for the penalized logistic regression (Elastic Net) model predicting POAF. The model retains measurable net clinical benefit across threshold probabilities between 0.15 and 0.55 compared with “treat all” and “treat none” strategies. Analysis performed on out-of-fold predictions obtained from nested stratified cross-validation.

Figure 3 presents the Decision Curve Analysis (DCA) for the penalized logistic regression model. The model demonstrates a consistent positive net clinical benefit across a broad range of threshold probabilities (approximately 0.15–0.55) compared with “treat-all” and “treat-none” strategies. This suggests that utilizing the model to inform postoperative rhythm monitoring and preventive management may yield superior clinical outcomes within realistic risk thresholds. The curve is based exclusively on out-of-fold (OOF) predictions from nested cross-validation, confirming internal robustness of the net benefit estimation.

As an additional sensitivity analysis, a constrained XGBoost model was trained under the same nested CV and calibration protocol (no oversampling; class imbalance handled via scale_pos_weight). Its OOF performance was comparable (AUC = 0.968 [95% CI 0.928–0.994], Brier = 0.054) and confirmed consistent directional effects across variables. Calibration and decision-curve analyses are shown in Supplementary Figures S1–S3. Collectively, these results demonstrate that a small set of a priori biatrial functional parameters can accurately discriminate POAF risk in this single-center cohort, with strong internal validity and satisfactory calibration. At the same time, all conclusions remain limited to internal validation pending external replication. To further illustrate model interpretability, SHAP analysis was performed on the full-fit XGBoost model (Supplementary Figure S4). Although exploratory and not used for variable selection, the SHAP summary plot revealed a directionally consistent ranking of predictors—TACT and MIN LA BSA exerted the most substantial positive contributions to POAF risk, while RAAEF and TAPSE contributed negatively. This alignment between the tree-based and penalized regression models reinforces the mechanistic plausibility of the identified biatrial dysfunction pattern.

## 4. Discussion

This study integrates echocardiographic and clinical data, utilizing both conventional statistics and interpretable machine-learning analysis, to examine the determinants of postoperative atrial fibrillation (POAF) after cardiac surgery. The findings indicate that POAF arises from the interplay of atrial conduction delay, biatrial volumetric and functional remodeling, and altered mitral valve mechanics—forming an electromechanical substrate that precedes the arrhythmic event. From a statistical standpoint, total atrial conduction time (TACT) emerged as the most informative single predictor, consistent with previous evidence that prolonged atrial activation reflects diffuse fibrotic remodeling and slowed intra-atrial conduction [15,29,30]. This electrical disorganization, particularly under postoperative inflammatory or hemodynamic stress, may promote macro-reentrant circuits and ectopic activity. In parallel, right atrial volumetric indices (minimal volume, indexed minimal volume, and pre-atrial volume) were associated with POAF risk, suggesting that right-sided mechanical distension contributes to stretch-induced electrical instability by impairing reservoir and booster-pump function [31].

The penalized logistic regression (Elastic Net) model provided a parsimonious and physiologically coherent framework for identifying these determinants. By integrating a limited set of a priori echocardiographic predictors—TACT, RAAEF, MIN LA BSA, and TAPSE—the model captured both temporal and mechanical aspects of atrial dysfunction. Regularization reduced multicollinearity and enhanced stability despite the modest number of events. Among the included parameters, prolonged TACT remained the dominant marker of conduction heterogeneity. In contrast, lower RAAEF and reduced TAPSE indicated compromised right atrial and right ventricular mechanics, highlighting the importance of atrioventricular interaction. Increased MIN LA BSA reflected left atrial structural remodeling and loss of compliance, emphasizing that POAF risk emerges from biatrial rather than unilateral dysfunction. The model demonstrated strong internal discrimination and satisfactory calibration based on nested out-of-fold predictions, representing internal validation only. These findings delineate a pilot-level analytical framework that characterizes the electromechanical profile of postoperative atrial vulnerability without implying clinical deployability. Future multicenter studies with larger cohorts will be required to assess external validity and integrate strain-derived parameters for enhanced mechanistic resolution [13,32].

Comparison of the penalized regression and machine-learning frameworks revealed consistent patterns of association. Both the Elastic Net and XGBoost models identified the same core echocardiographic predictors—TACT, RAAEF, MIN LA BSA, and TAPSE—as the dominant features. While the penalized regression quantified linear contributions and yielded interpretable coefficients, XGBoost explored nonlinear and hierarchical interactions among conduction and mechanical parameters. The concordance between the two modeling approaches suggests that these associations are intrinsic to the dataset rather than artifacts of specific methodology. The accompanying SHAP analysis (Supplementary Figure S4) was used exclusively for exploratory interpretability and confirmed that the relative feature importance mirrored that of the penalized regression model. This alignment between linear and tree-based analyses enhances confidence in the physiological plausibility of the observed electromechanical relationships while avoiding causal inference [33].

The present findings align with and extend the existing literature, which describes POAF as a multifactorial electromechanical disorder rather than an isolated electrical event. Across surgical cohorts, prolonged TACT has been validated as a noninvasive marker of atrial remodeling and arrhythmic vulnerability, correlating with histologic fibrosis and conduction reserve impairment [15]. Our results support its central role in the arrhythmogenic substrate. Complementary evidence highlights the contribution of right atrial loading and mechanical dysfunction to postoperative arrhythmogenesis, which is consistent with our observation that impaired RA mechanics and reduced RV–tricuspid coupling accompany POAF [31,34]. This underscores that atrial vulnerability reflects not only left-sided remodeling but also systemic and right-sided mechanical interplay.

Left ventricular diastolic dysfunction and impaired atrioventricular coupling have also been repeatedly linked to POAF through elevated atrial pressure and stretch [32,35]. The observed association between E/A-based indices and POAF is consistent with this pathophysiologic mechanism, emphasizing the role of ventricular compliance in modulating atrial electrophysiologic stability. Furthermore, imaging studies have shown that left atrial strain improves discrimination over size alone, supporting a functional rather than morphological paradigm of risk [13]. Similarly, our analysis indicates that indices reflecting dynamic atrial function may better characterize postoperative arrhythmic vulnerability than static dimensions.

With respect to clinical risk stratification, the higher HATCH scores in the POAF group align with prior evidence that conventional risk tools provide only moderate discrimination compared to imaging-enriched models [33]. Likewise, the observed relationship between chronic obstructive pulmonary disease (COPD) and POAF aligns with previous reports identifying COPD as an independent risk factor mediated by hypoxemia, pulmonary hypertension, and diastolic loading [36,37]. Collectively, these data situate POAF within a coherent pathophysiologic triad: electrical conduction delay, biatrial mechanical overload, and impaired ventricular–atrial coupling.

Recent studies employing interpretable machine-learning algorithms (e.g., gradient boosting with SHAP) have demonstrated the feasibility of integrating echocardiographic and clinical variables for POAF risk modeling [33,38]. Our exploratory ML results are consistent with this literature but are intentionally limited to internal validation. Such methods are valuable for hypothesis generation, but their generalizability critically depends on external datasets and prospective evaluation.

Although not assessed in this cohort, speckle-tracking echocardiography (STE)–derived left atrial reservoir strain (LASr) has recently gained recognition as a sensitive marker of atrial mechanical remodeling and a strong predictor of atrial fibrillation recurrence. Over the past decade, several studies have demonstrated that LASr provides incremental prognostic information beyond conventional Doppler and volumetric indices in identifying patients at elevated risk of postoperative or recurrent AF. In a prospective study by Sarvari et al., reduced atrial deformation and increased mechanical dispersion, as measured by STE, independently predicted AF recurrence after ablation, even in patients with preserved atrial size [39]. Similarly, Vincenti et al. showed that impaired LA strain after cardioversion reflected incomplete mechanical recovery and higher recurrence risk, underscoring the sensitivity of strain imaging to dynamic atrial remodeling [40]. A recent systematic review by Barilli et al. confirmed the prognostic value of LASr, particularly in patients with paroxysmal AF and in low-risk populations, and highlighted its potential role in individualized risk stratification [41]. Although strain data were unavailable for all patients in the present cohort, the consistency of these reports supports the same mechanistic framework proposed here—namely, that functional atrial impairment precedes and predicts arrhythmic vulnerability. Future prospective studies incorporating STE-derived strain parameters are warranted to refine and externally validate the current electromechanical model.

Several limitations must be acknowledged. This was a single-center retrospective study, which inherently restricts external generalizability and carries potential for unmeasured confounding. Although the cohort was homogeneous regarding surgical technique and perioperative management, it may not fully represent the broader cardiac surgery population. The sample size, though adequate for penalized regression, remains modest for complex ML models, and the observed high internal performance should be interpreted cautiously. External validation on independent data will be essential to confirm reproducibility. Echocardiographic measurements, such as TACT, atrial volumes, and Doppler parameters, depend on image quality and operator technique, and vendor-specific differences may affect the derived values. Moreover, the study relied on two-dimensional and Doppler modalities, whereas advanced techniques such as three-dimensional imaging or strain analysis could provide deeper mechanistic insights.

The models focused on preoperative and early perioperative factors; dynamic postoperative influences such as inflammation, electrolyte imbalance, or autonomic variation were not analyzed. Biochemical and electrophysiological data (e.g., oxidative stress markers, fibrosis indices, continuous rhythm monitoring) were unavailable, limiting mechanistic interpretation. Methodologically, both regression and ML frameworks are data-driven and non-causal. While interpretability methods improve transparency, their outputs must be contextualized within established physiological knowledge.

In summary, this study provides an internally validated depiction of the electromechanical determinants of POAF, demonstrating convergence between conventional and machine-learning analyses. The results should be viewed as exploratory and hypothesis-generating, aimed at informing future prospective, multicenter, and multimodal investigations that will determine clinical applicability and external validity of this modeling approach.

## 5. Conclusions

In conclusion, this study identifies a distinctive electromechanical phenotype that predisposes patients to POAF following cardiac surgery. By integrating echocardiographic and clinical parameters through both classical statistical and advanced machine-learning approaches, we demonstrated that POAF risk is governed by the interaction between atrial conduction delay (TACT), biatrial mechanical remodeling, and mitral valve coupling dynamics. Among these, TACT emerged as the most consistent and physiologically meaningful predictor, serving as a unifying marker of atrial electrical and structural vulnerability. The combination of conventional regression and interpretable XGBoost–SHAP analysis provided complementary perspectives, confirming linear relationships while uncovering nonlinear dependencies across the conductive, volumetric, and valvular domains.

The results highlight that POAF is not merely a transient postoperative complication but the clinical manifestation of a pre-existing electromechanical disequilibrium that can be objectively quantified before surgery. This recognition carries significant implications for risk stratification and early intervention: echocardiographic metrics of atrial conduction and atrioventricular coupling may serve as feasible, reproducible markers to identify patients at heightened arrhythmic risk. Moreover, explainable artificial intelligence provides a novel framework for translating these multidimensional signatures into individualized predictions, thereby bridging the gap between advanced imaging and precision perioperative management.

Future multicenter and prospective studies are warranted to externally validate the proposed predictive architecture, define clinically actionable thresholds, and explore whether targeted modulation of atrial conduction or mechanical loading can mitigate the burden of POAF. Together, these findings establish a mechanistically coherent and data-driven foundation for a new generation of risk assessment tools that integrate echocardiographic physiology, machine learning, and clinical decision support in cardiac surgery.

## Figures and Tables

**Table 1:** Baseline demographic, clinical, and echocardiographic characteristics of patients with and without postoperative atrial fibrillation (POAF).

Variable	No-POAF	POAF	*p*-Value
Age (years)	63.86 ± 7.91	68.70 ± 7.64	0.0009
PR interval (ms)	164.23 ± 27.89	174.28 ± 22.23	0.0257
Maximal LA (mL)	36.08 ± 9.07	39.09 ± 7.26	0.0439
Minimal LA (mL)	39.62 ± 15.81	54.48 ± 13.05	<0.001
Minimal LA/BSA (mL/m^2^)	20.73 ± 8.55	28.19 ± 6.52	<0.001
Pre-A LA/BSA (mL/m^2^)	27.37 ± 8.20	31.17 ± 6.91	0.0067
Transmitral E (m/s)	0.81 ± 0.23	0.59 ± 0.15	<0.001
Transmitral A (m/s)	0.84 ± 0.20	0.42 ± 0.25	<0.001
E/A ratio	1.00 ± 0.36	1.78 ± 0.69	<0.001
RA active emptying fraction (RAAEF)	0.28 ± 0.12	0.11 ± 0.05	<0.001
TAPSE (mm)	20.09 ± 3.09	16.78 ± 4.17	<0.001
Creatinine clearance (mL/min)	81.03 ± 22.46	72.72 ± 19.25	0.031
Neutrophils (%)	69.15 ± 15.92	75.99 ± 12.36	0.0073
HATCH = 2	9.50%	38.30%	0.0014
NYHA ≥ III	11%	32%	0.003
COPD present	12% (yes)	31% (yes)	0.0068
Smoker	18% (yes)	39% (yes)	0.0153
Intra-operative amiodarone	10% (yes)	27% (yes)	0.0153
NIV required	7%(yes)	25% (yes)	0.0281
Re-intubation	3% (yes)	17% (yes)	0.0127

Abbreviations: LA—left atrium; RA—right atrium; BSA—body surface area; E/A—early-to-late diastolic transmitral flow velocity ratio; RAAEF—right atrial active emptying fraction; TAPSE—tricuspid annular plane systolic excursion; PR—PR interval on electrocardiogram; HATCH—Hypertension, Age ≥ 75 years, Transient ischemic attack or stroke, Chronic obstructive pulmonary disease, Heart failure score; NYHA—New York Heart Association functional class; COPD—chronic obstructive pulmonary disease; NIV—non-invasive ventilation; CrCl—creatinine clearance; E—early diastolic transmitral flow velocity; A—late diastolic transmitral flow velocity.

**Table 2:** Univariate logistic regression analysis of clinical and echocardiographic predictors of POAF.

Age (Years)	OR	95% CI (Lower–Upper)	*p*-Value	Direction
TACT	1.276	1.140–1.427	<0.001	↑ Risk
MIN RA BSA	1.441	1.287–1.614	<0.001	↑ Risk
Minimal RA	1.219	1.143–1.300	<0.001	↑ Risk
PRE A RA BSA	1.32	1.198–1.453	<0.001	↑ Risk
MIN LA BSA	1.124	1.064–1.187	<0.001	↑ Risk
E/A ratio	13.709	5.406–34.761	<0.001	↑ Risk
TAPSE	0.766	0.678–0.865	<0.001	↓ Risk
KEDA	0.026	0.007–0.100	<0.001	↓ Risk
Age (years)	1.093	1.034–1.156	0.0017	↑ Risk
NYHA = IV	5.417	1.774–16.546	0.003	↑ Risk
COPD	3.469	1.409–8.542	0.0068	↑ Risk
Smoking	2.476	1.190–5.151	0.015	↑ Risk
Intra-operative amiodarone	2.476	1.190–5.151	0.015	↑ Risk
HATCH = 2	5.897	2.312–15.039	<0.001	↑ Risk

Values are presented as odds ratios (OR) with 95% confidence intervals (CI). Arrows indicate the direction of association with POAF (↑—increased risk; ↓—protective effect). Abbreviations: TACT—total atrial conduction time; RA—right atrium; LA—left atrium; BSA—body surface area; TAPSE—tricuspid annular plane systolic excursion; KEDA—kinetic energy of the left atrium; NYHA—New York Heart Association functional class; COPD—chronic obstructive pulmonary disease; HATCH—Hypertension, Age ≥ 75 years, Transient ischemic attack or stroke, Chronic obstructive pulmonary disease, Heart failure score.

**Table 3:** Multivariable penalized logistic regression (Elastic Net) based on four a priori echocardiographic predictors of POAF.

Predictor	Penalized β	OR (Approx.)	95% CI (Bootstrap)	Direction
TACT (ms)	0.78	2.18	1.14–4.10	↑ Risk
RAAEF (%)	−0.65	0.52	0.28–0.96	↓ Risk
MIN LA BSA (mL/m^2^)	0.6	1.82	1.02–3.26	↑ Risk
TAPSE (mm)	−0.71	0.49	0.25–0.90	↓ Risk

Arrows indicate the direction of association with POAF (↑—increased risk; ↓—protective effect). TACT—total atrial conduction time (ms); RAAEF—right atrial active emptying fraction (%); MIN LA BSA—minimal left atrial volume indexed to body surface area (mL/m^2^); TAPSE—tricuspid annular plane systolic excursion (mm).

## Data Availability

The data that support the findings of this study are available upon request from the corresponding author.

## Supplementary Materials

The following supporting information can be downloaded at https://www.mdpi.com/article/10.3390/medicina61112038/s1. Figure S1: Receiver Operating Characteristic (ROC) curve for the XGBoost model obtained through nested stratified cross-validation. The model demonstrated excellent internal discrimination (AUC = 0.967 [95 % CI 0.928–0.994]). All values are based on out-of-fold (OOF) predictions to avoid optimistic bias. Figure S2: Calibration curve for the XGBoost model (nested cross-validation, OOF predictions). The observed frequencies of postoperative atrial fibrillation (POAF) closely followed the predicted probabilities, indicating satisfactory internal calibration after Platt scaling. Minor deviations in higher probability deciles reflect the limited number of positive events. Figure S3: Decision Curve Analysis (DCA) for the XGBoost model. The net benefit curve (blue) demonstrates consistent superiority over “treat all” (red dashed) and “treat none” (gray dashed) strategies for threshold probabilities between approximately 0.15 and 0.60. Analysis performed on out-of-fold predictions from nested cross-validation. Figure S4: SHAP summary plot for the full-data XGBoost model (exploratory, non-inferential). TACT and MIN LA BSA showed the strongest positive contributions to POAF risk, while RAAEF and TAPSE were associated with a lower risk. The figure is presented solely for illustrative purposes to highlight consistency with the penalized regression model; SHAP values were not used for variable selection.
