# Supplementary material for: Integrative Evaluation of Atrial Function and Electromechanical Coupling as Predictors of Postoperative Atrial Fibrillation
Source: Medicina (Kaunas). 2025 Nov 14;61(11):2038. doi: 10.3390/medicina61112038 (PMC12654043; doi:10.3390/medicina61112038)
Supplement: Supplementary file 1 [file medicina-61-02038-s001.zip › medicina-3953258-supplementary.pdf]

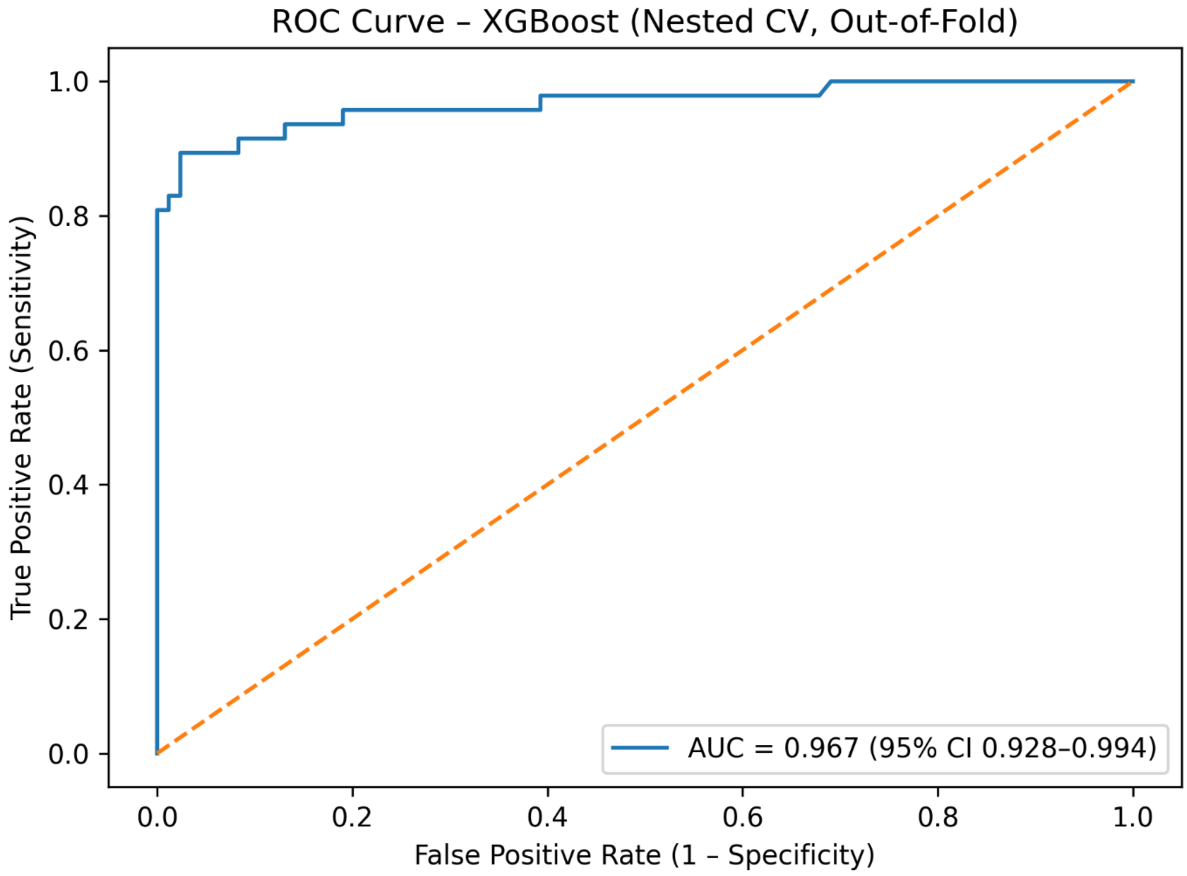

Figure S1. Receiver Operating Characteristic (ROC) curve for the XGBoost model obtained through nested stratified cross-validation. The model demonstrated excellent internal discrimination (AUC = 0.967 [95 % CI 0.928–0.994]). All values are based on out-of-fold (OOF) predictions to avoid optimistic bias.

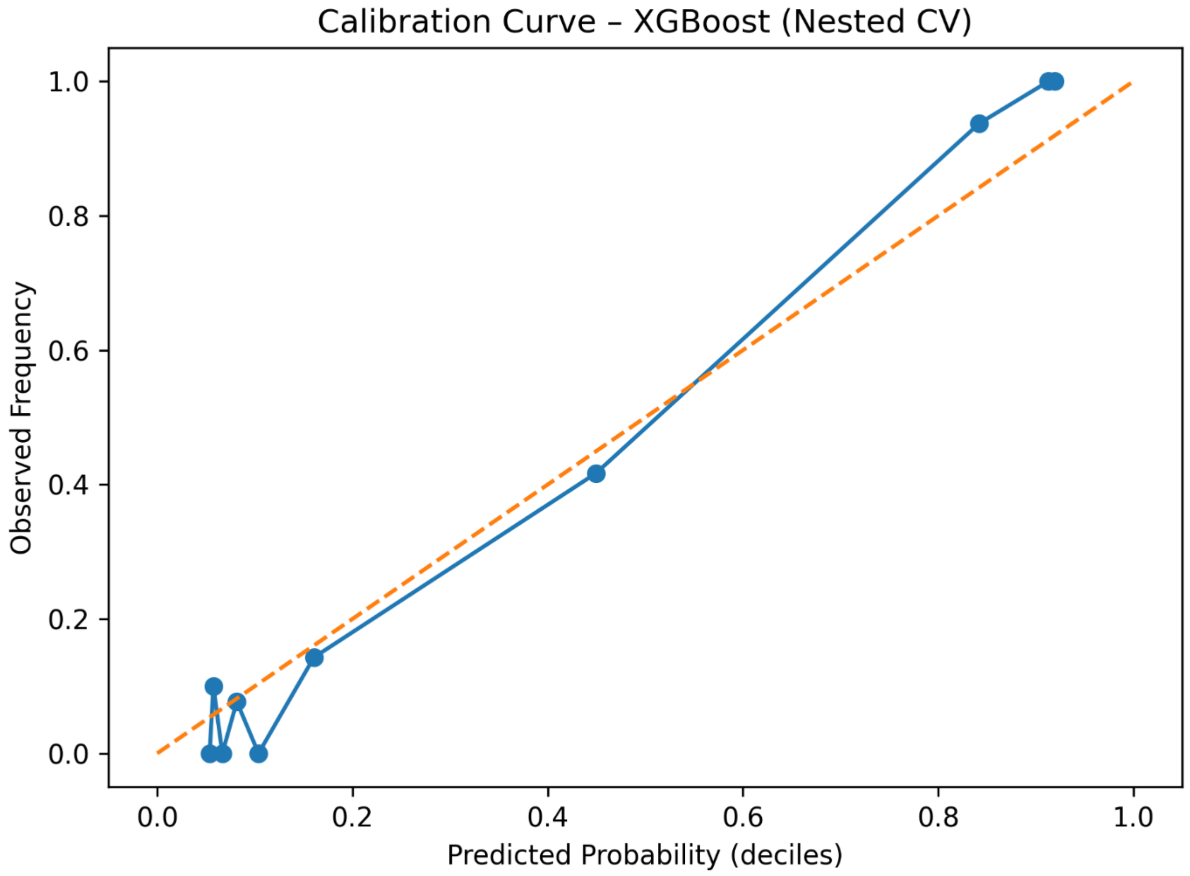

Figure S2. Calibration curve for the XGBoost model (nested cross-validation, OOF predictions). The observed frequencies of postoperative atrial fibrillation (POAF) closely followed the predicted probabilities, indicating satisfactory internal calibration after Platt scaling. Minor deviations in higher probability deciles reflect the limited number of positive events.

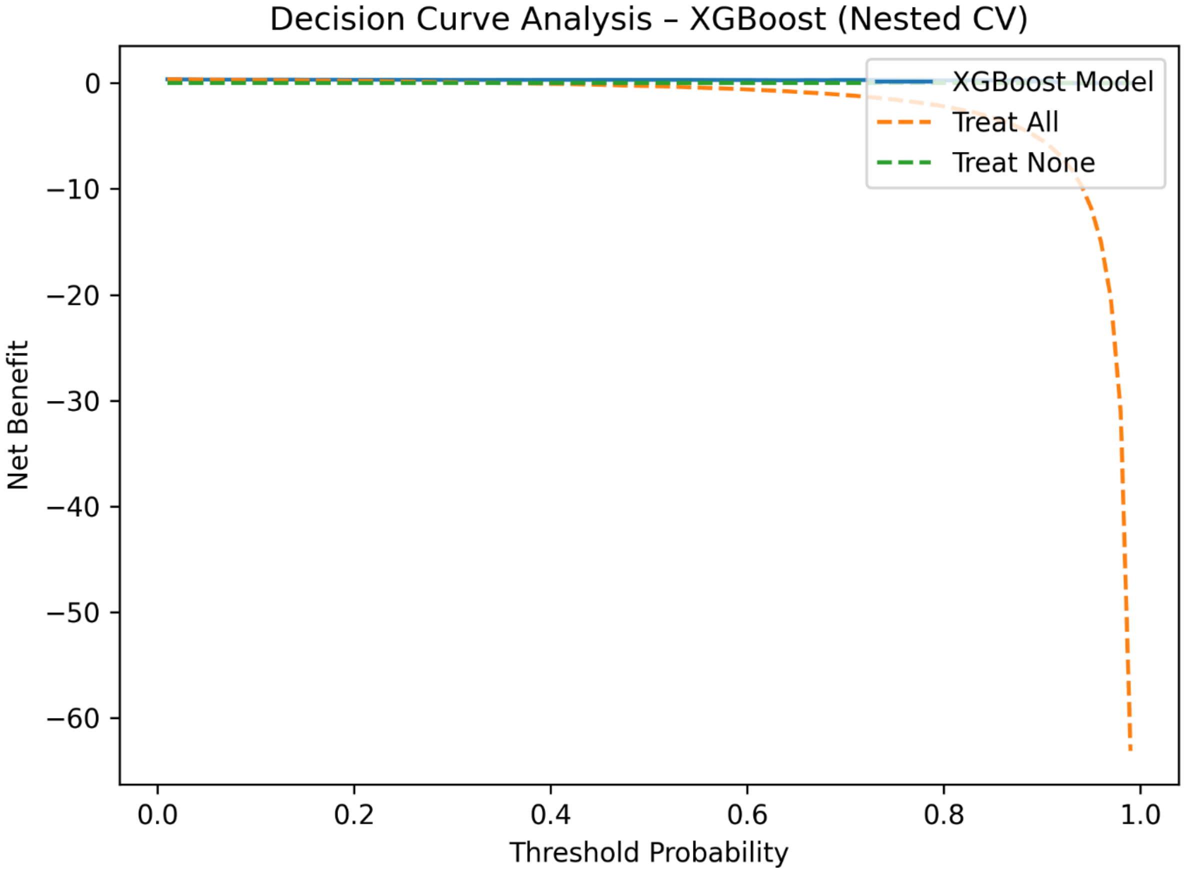

Figure S3. Decision Curve Analysis (DCA) for the XGBoost model. The net benefit curve (blue) demonstrates consistent superiority over "treat all" (red dashed) and "treat none" (gray dashed) strategies for threshold probabilities between approximately 0.15 and 0.60. Analysis performed on out-of-fold predictions from nested cross-validation.

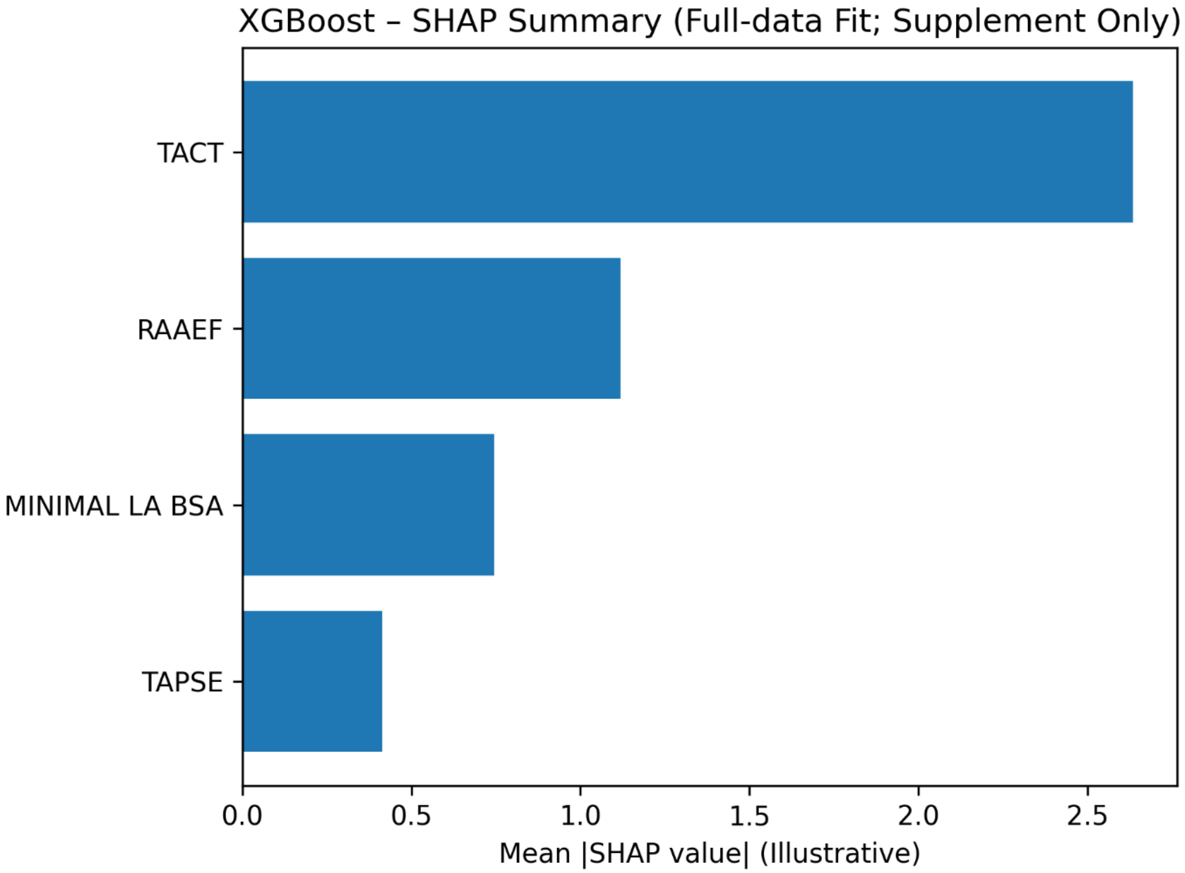

Figure S4. SHAP summary plot for the full-data XGBoost model (exploratory, non-inferential). TACT and MIN LA BSA showed the strongest positive contributions to POAF risk, while RAAEF and TAPSE were associated with a lower risk. The figure is presented solely for illustrative purposes to highlight consistency with the penalized regression model; SHAP values were not used for variable selection.
